# Impact of guided inquiry-based laboratory experiments on secondary school students’ attitudes toward biology in Ethiopia: A quasi-experimental study

**DOI:** 10.1371/journal.pone.0326278

**Published:** 2025-12-30

**Authors:** Ashebir Mekonnen Chengere, Beyene Dobo Bono, Samuel Assefa Zinabu, Kedir Woliy Jilo

**Affiliations:** 1 Department of Biology, College of Natural and Computational Science, Hawassa University, Hawassa, Ethiopia; 2 School of Education, College of Education, Hawassa University, Hawassa, Ethiopia; Zhejiang Normal University, CHINA

## Abstract

Attitude plays a vital role in achieving modern science education goals by enhancing students’ conceptual understanding, motivation, academic performance, interest, and engagement in scientific inquiry. However, in many Ethiopian schools, attitudes toward science are often overlooked, and traditional teacher-centered instruction combined with limited resources hampers their development. To address these issues, this study examined the effect of Guided Inquiry-Based Laboratory Experiments Enriched Instruction (GIBLEI) on secondary school students’ attitudes toward biology, using a quasi-experimental design with pretest-treatment-posttest phases. Two biology classes from purposively selected Ethiopian secondary schools were randomly assigned to an experimental group (EG, N = 46) and a control group (CG, N = 29). Over eight weeks, the EG received GIBLEI, while the CG experienced Traditional Laboratory Experiments Enriched Instruction (TLEI). Attitudes were measured using a 5-point Likert scale. The results showed that GIBLEI significantly improved students’ overall attitude toward biology, enthusiasm for biology, perception of biology as a course, and understanding of biology as a process, compared to TLEI. However, it did not significantly affect their views of biology as a career. GIBLEI also promoted gender inclusivity by reducing attitude differences between male and female students. These findings highlight the benefits of GIBLEI in fostering positive attitudes, engagement, and inclusivity in biology education, enhancing both student outcomes and equity in science learning.

## 1. Introduction

The development of scientifically literate individuals is indispensable for the advancement of societies, impacting not only their socio-economic fabric but also the scientific progress of societies [1]. Consequently, significant attention has been directed toward enhancing science education, particularly at the secondary level, across various nations. Notably, countries in Sub-Saharan Africa, including Ethiopia, have embarked on curriculum reforms, transitioning from knowledge-centric to competence-based approaches. This shift aims to equip learners with the essential scientific attitudes, skills, and values that promote effective science learning and contribute to building a sustainable future [2].

Attitudes represent a person’s opinions, beliefs, and emotional dispositions influencing how they perceive and respond to scientific ideas and experiences [3]. When students hold positive attitudes toward science, they become more motivated and actively engaged in learning. Such attitudes enhance their conceptual understanding and overall academic performance. Consequently, fostering positive attitudes toward science encourages deeper interest and sustained participation in scientific learning [4].

In Ethiopia, despite the national science curriculum setting out well-defined and ambitious learning goals, the actual delivery of science education in many secondary schools remains below expectations. In other words, there is a noticeable gap between the intended curriculum and the way science is taught and learned in practice. Research on the Ethiopian educational institutions revealed that the attitude of students toward science learning including biology is declining along with their academic achievement [5]. As indicated in the Ethiopian Education Development Roadmap (2018−30), a majority of the students (55.1%) perceived the teaching-learning process in their respective secondary schools as either partly uninteresting (34.3%) or as completely boring (20.8%) [6].

Moreover, although laboratory work is considered an essential part of science education, its actual use in many Ethiopian secondary schools frequently fails to achieve the desired educational outcomes [7]. It is expected to play a crucial role in enhancing student engagement and improving attitudes toward science including biology by offering hands-on, experiential learning that connects abstract concepts to real-world experiences [8]. However, many biology classrooms still rely on teacher-centered, traditional laboratory methods, where students follow rigid, step-by-step instructions with predetermined outcomes, limiting creativity and opportunities for independent thinking. To improve students’ attitudes toward biology, it is essential to transition from these traditional methods to more student-centered, inquiry-driven approaches that foster autonomy, engagement, and critical thinking. Additionally, addressing gender biases and promoting inclusivity in science education can further enhance students’ attitudes by creating a more equitable learning environment, where all students, especially girls, feel empowered to pursue their interests in biology and other STEM fields [9].

Inquiry-Based Learning (IBL) has emerged as a transformative pedagogical strategy that improves students’ attitudes toward science subjects, including biology. By placing students at the center of the learning process, IBL encourages them to ask questions, explore real-world problems, construct their own knowledge, and reflect on their learning [10]. This active engagement fosters a sense of curiosity and ownership, which contributes to more positive attitudes towards the subject matter. Several studies have demonstrated that IBL can increase students’ interest, enjoyment, and motivation in learning biology by making the subject more relevant, interactive, and intellectually stimulating. Moreover, hands-on, inquiry-based laboratory activities allow learners to connect theoretical concepts to practical experiences, which has been shown to reduce negative perceptions and anxiety associated with science learning [11]. In biology education specifically, IBL has been associated with increased enthusiasm and engagement, which are key dimensions of student attitude [12].

However, implementing Inquiry-Based Learning (IBL) in resource-constrained contexts like Ethiopia presents several challenges that hinder its effectiveness in fostering positive student attitudes toward biology. Key barriers include inadequate laboratory facilities, limited access to instructional materials, and a shortage of teachers trained in inquiry methodologies [13]. These constraints make it difficult to conduct hands-on, exploratory activities central to IBL, often causing students to view biology as abstract and disconnected from real life. Additionally, the open-ended nature of IBL may overwhelm students who lack foundational knowledge or prior exposure to student-centered pedagogies, potentially leading to confusion, frustration, and decreased interest [14]. Teachers, many of whom are accustomed to traditional, lecture-based methods, may resist implementing IBL due to limited professional development and discomfort with facilitating student-directed learning. In environments where students are unaccustomed to taking initiative, the absence of scaffolding can further weaken engagement and hinder the development of favorable attitudes toward biology [10].

In response to the limitations of IBL, this study proposes a Guided Inquiry-Based Laboratory Experiments Enriched Instructional (GIBLEI) approach, an adapted form of guided inquiry based learning (GIBL) designed for secondary schools with limited resources. To overcome these limitations, GIBL is increasingly recommended in biology education because it strikes a balance between open-ended exploration and structured support. Unlike fully open inquiry, which may overwhelm students with little prior experience, guided inquiry scaffolds learning by offering clear procedural guidance while still engaging students in core scientific practices, such as formulating hypothesis, investigating, interpreting evidence, and drawing conclusions [10].

Moreover, although Inquiry-Based Learning (IBL) and its variations, such as Guided Inquiry-Based Learning (GIBL), have been widely recognized for improving students’ science learning outcomes across the world, their implementation and impact remain insufficiently explored in developing countries like Ethiopia [15]. Most existing studies have focused on general inquiry-based instruction rather than laboratory-centered approaches that integrate inquiry processes with hands-on experimentation. To date, no empirical research has examined the impact of the GIBLEI approach on enhancing students’ attitudes toward biology within the Ethiopian secondary school context.

Thus, this study investigated the impact of the GIBLEI approach on secondary school students’ attitude toward biology compared to the Traditional Laboratory Experiments Enriched Instructional (TLEI) approach in North Shoa Zone, Oromia Regional State, Ethiopia. Specifically, it examines students’ attitudes across four key dimensions: enthusiasm for biology, perceptions of biology learning as a subject, understanding of biology as a process, and interest in biology as a career. It is expected that students taught using the GIBLEI will show higher post-test attitude scores than those taught using TLEI. Null hypotheses were formulated to assess the effects of the instructional methods and determine whether significant differences exist between the experimental group (EG) and control group (CG).

***H***₀_***1***_**:** There is no statistically significant difference in the overall post-test mean scores of students’ attitudes toward biology between the GIBLEI and TLEI groups.

***H***₀_***2***_**:** There are no statistically significant differences in the post-test mean scores on the four attitude dimensions (enthusiasm, biology learning, biology as a process, and biology as a career) between the GIBLEI and TLEI groups.

***H***₀_***3***_**:** There is no statistically significant difference between the pre-test and post-test mean scores of overall attitude within each group.

***H***₀_**4**_: There are no statistically significant differences in the post-test mean scores of overall attitude and the four attitude dimensions between male and female students within each group.

This study was guided by the following research questions:

**RQ**_**1**_: Does a statistically significant difference exist in the overall post-test mean scores of students’ attitudes toward biology between those taught using the GIBLEI approach and those taught using the TLEI approach?

**RQ**_**2**_: Are there statistically significant differences in the post-test mean scores of the four specific attitude dimensions: enthusiasm, biology learning, biology as a process, and biology as a career, between the EG and CG?

**RQ**_**3**_: Within each instructional group, is there a statistically significant difference between students’ pre-test and post-test mean scores for overall attitude toward biology?

**RQ**_**4**_: Are there statistically significant gender-based differences in the post-test mean scores for overall attitude and the four attitude dimensions among male and female students within each group?

## 2. Materials and methods

### 2.1. Research design

This study examined the impact of the Guided Inquiry-Based Laboratory Experiments Enriched Instructional (GIBLEI) approach on grade 10 students’ attitudes toward biology. A total of 75 students (35 boys and 40 girls) from two public secondary schools in Fitche Town, North Shoa Zone, Ethiopia, participated in the study. The experimental group (n = 46), drawn from Fitche Secondary School, received instruction through the GIBLEI approach, while the control group (n = 29), from Abdisa Aga Secondary School, was taught using the Traditional Laboratory Experiments Enriched Instructional (TLEI) method. The intervention targeted Unit 4 of the grade 10 biology curriculum, ‘*Food Making and Growth in Plants’*, which includes plant organs, photosynthesis, transport, and response mechanisms, topics often associated with persistent student misconceptions [16].

A non-equivalent quasi-experimental pretest–posttest design was employed. This design is appropriate for educational contexts where random assignment is impractical due to the use of intact classrooms. To ensure group comparability, the selected schools were matched based on the similarity of their facilities and teacher qualifications. A pretest was administered to both groups using a validated Biology Attitude Questionnaire, which assessed students’ overall attitude toward biology and four specific dimensions: enthusiasm, biology learning as a subject, understanding of biology as a process, and interest in a biology-related career. Responses were recorded on a 5-point Likert scale (ordinal level). An independent samples t-test comparing pretest scores between the experimental and control groups showed no statistically significant differences (p > 0.05) for either the overall attitude or any of the four dimensions. This result demonstrates baseline equivalence between the groups, reducing potential selection bias and thereby strengthening the internal validity of the design.

### 2.2. Samples and sampling technique

The study was conducted in the North Shoa Zone of the Oromia Regional State in Ethiopia, focusing on grade ten students enrolled in public secondary schools during the 2023 academic year**.** A multistage sampling technique was employed to select the study participants and schools. Two public secondary schools, Fitche Secondary School and Abdisa Aga Secondary School**,** were selected using purposive sampling**.** Purposive sampling was employed to ensure that the selected schools had comparable infrastructure, student populations, and staff qualifications, thereby minimizing extraneous variability that could influence the outcomes**.** Specifically, the schools were chosen based on the similarity of their laboratory facilities, availability of laboratory chemicals and equipment, library resources, ICT access, and the qualifications and teaching experience of biology teachers. This careful selection helped control for contextual disparities that might otherwise confound the study results.

The selected schools were then randomly assigned to serve as either the experimental group (EG) or the control group (CG) using a simple lottery method. Fitche Secondary School was designated as the EG, while Abdisa Aga Secondary School served as the CG. To ensure instructional consistency, two qualified and experienced biology teachers, one from each school, were purposively selected to participate in the study. These teachers had a minimum of a Master’s degree in Biology or Biology Education and more than five years of teaching experience**,** ensuring comparable teaching competence. Subsequently, one intact class from each school was selected using a simple random sampling (lottery) method. The class from Fitche Secondary School, consisting of 46 students (22 males and 24 females), was assigned to the experimental group (EG), while the class from Abdisa Aga Secondary School consisting of 29 students (13 males and 16 females), was assigned to the control group (CG). The selection of intact classes was based on the need to maintain the natural classroom setting and minimize disruption to normal teaching schedules. Overall, 75 grade ten students (35 boys and 40 girls) participated in the study. The sample size was determined by the natural class sizes available in the selected schools and is consistent with previous quasi-experimental studies in similar contexts [17,18], which typically involve 60–100 participants to achieve adequate statistical power for detecting medium effect sizes.

Moreover, only regularly attending students who provided informed consent were included. Students who had not yet received formal instruction on the selected topic (“Food Making and Growth in Plants”) were eligible for participation. Those who missed more than 25% of the intervention sessions, transferred schools, or withdrew before completing the post-test were excluded from the analysis.

The study focused on Unit Four of the Grade 10 biology curriculum, titled “Food Making and Growth in Plants,” which includes key subtopics such as the organs of flowering plants, leaves, photosynthesis, transport, and plant response mechanisms. This unit was purposively selected due to its pedagogical significance and the central role of photosynthesis in understanding plant biology. The topic of photosynthesis, in particular, was chosen because it is both foundational and conceptually challenging, making it ideal for evaluating different instructional approaches. Numerous studies have documented common student misconceptions, such as the belief that plants consume soil to gain nutrients and produce their entire biomass [19], or that plants require only carbon dioxide while confusing photosynthesis with respiration [16]. These persistent misunderstandings provided a meaningful and appropriate context for assessing the effectiveness of the GIBLEI approach compared to traditional instruction.

### 2.3. Data collection instrument

The Biology Attitude Questionnaire (BAQ) was adapted from Kaur & Zhao [20] to assess secondary school students’ attitudes toward biology. The questionnaire, consisting of 36 items, was administered both before and after the intervention to capture changes in students’ perceptions. A five-point Likert scale measured attitudes across four dimensions: enthusiasm toward biology, biology learning as a subject, biology as a process, and biology as a career. Responses were recorded on a five-point Likert scale ranging from 1 (strongly disagree) to 5 (strongly agree).with positive items scored directly and negative items reverse-scored to maintain consistency. Mean scores for each dimension were calculated by averaging responses to the related statements, allowing for detailed insights into students’ attitudes.

The first dimension, enthusiasm toward biology, includes seven statements reflecting students’ feelings toward the subject, ranging from enjoyment and importance (positive extreme) to aversion (negative extreme). The second dimension, biology learning as a subject, encompasses 11 statements that explore students’ perspectives on discovery, comprehension, and active engagement (positive views) versus formal, limited, and mechanical learning (negative views). The third dimension, biology as a process, contains nine statements addressing the students’ understanding of biology as a dynamic, evolving discipline (positive view) versus a rigid, nearly complete field (negative view). Lastly, the fourth dimension, biology as a career, includes nine statements assessing perceptions of biologists and interest in biology-related careers, contrasting positive views of biologists as competent professionals with negative stereotypes of them as antisocial or eccentric. By averaging mean scores across all 36 items, the overall attitude toward biology was determined, while individual dimension scores provided a more granular understanding of students’ perspectives. This structured approach facilitated a comprehensive analysis of changes in attitudes, offering insights into how students perceive biology and its relevance to their lives and future aspirations.

#### 2.3.1. Validity of the Biology Attitude Questionnaire (BAQ).

Validity refers to the extent to which an instrument measures what it is intended to measure [21]. In this study, emphasis was placed on content and face validity to ensure that the BAQ accurately captured students’ attitudes toward biology. The BAQ was adapted from the Physics Attitude Questionnaire developed by Kaur & Zhao [20], with terminology changed from physics to biology and irrelevant items removed. The original 60-item scale, which addressed five dimensions, Enthusiasm toward Physics, Physics Learning, Physics as a Process, Physics Teacher, and Physics as a Future Vocation, was refined in collaboration with two experienced biology teachers and two PhD students specializing in biology education. This process streamlined the instrument to 36 items grouped into four dimensions: Enthusiasm toward Biology, Biology learning as a subject, Biology as a Process, and Biology as a Career. The four experts, two PhD students and two biology teachers, rated the relevance of each item using a 5-point scale (1 = “not relevant”, 2 = “slightly relevant,” 3 = “moderately relevant,” 4 = “very relevant,” and “5 = “highly relevant”). The resulting Aiken’s V coefficients ranged from 0.78 to 0.91, all exceeding the 0.70 threshold for acceptable content validity, indicating strong agreement among experts on the relevance of all items [22].

Face validity was assessed by the same panel of experts through a structured review of the BAQ. Printed copies were provided, and experts evaluated each item for clarity, simplicity, and cultural appropriateness for Ethiopian secondary school students. They also judged whether the items appeared to measure attitudes toward biology effectively and flagged any ambiguities or potential misunderstandings. Feedback was discussed in group sessions, and items were revised collaboratively to enhance clarity and alignment with the intended constructs. This process ensured that the questionnaire was perceived as relevant, understandable, and appropriate for the target audience.

To ensure accessibility for all participants, the BAQ was translated into Amharic and Afan Oromo by professional language experts. A back-translation procedure was then carried out, whereby independent translators retranslated the items into the original language. The back-translated versions were compared with the original questionnaire to detect and resolve any discrepancies in meaning. Experts in psychology and language studies reviewed the translations to confirm their accuracy, clarity, and cultural relevance. This rigorous translation and validation process ensured that the BAQ maintained its intended meaning across languages and was suitable for diverse linguistic backgrounds within the Ethiopian context.

#### 2.3.2. Reliability of the BAQ.

Internal consistency reliability, commonly measured by Cronbach’s coefficient alpha, assesses how well a set of items measures a single construct [23]. In this study, Cronbach’s alpha was used to evaluate the internal consistency of the Biology Attitude Questionnaire (BAQ). The pilot study yielded an overall alpha coefficient of 0.834, indicating good reliability. However, items 2, 16, 22, and 28 were removed following reliability analysis, as they reduced the internal consistency of the scale.After these adjustments, the revised BAQ demonstrated reliable internal consistency across its four attitude dimensions: “Enthusiasm” (α = 0.701), “Biology Learning” (α = 0.703), “Biology as a Process” (α = 0.707), and “Biology as a Career” (α = 0.704). These alpha values fall within the acceptable range, suggesting the questionnaire is reliable for capturing attitudes toward biology. The four removed items were carefully reviewed to address issues of internal consistency and ensure they met reliability standards. After this review and refinement, these items were integrated into the improved version of the Biology Attitude Questionnaire (BAQ). The finalized version of the BAQ, incorporating these adjustments, was validated and prepared for the actual data collection phase, ensuring it served as a reliable and robust tool for assessing student attitudes toward biology.

### 2.4. The treatment procedures

#### 2.4.1. Pre-treatment procedure.

In the 1^st^ stage, the teacher and the laboratory technician from the EG were given training on the instructional and training materials prepared by the researchers. The training included a detailed description of the GIBLEI. It also encompassed how to use the 5E lesson plan format to prepare a daily lesson plan by teachers. The training was given by the researchers and lasted for 3 days, 90 minutes per day. On the first day, 90 minutes were devoted to explaining the materials, while the remaining days were used for classroom practice. In addition, orientation was given on the aim of the research to the teacher and laboratory technician from the CG before intervention. Then, the BAQ pretest was administered to the two groups. After the completion of all the preliminary activities, intervention was started. The intervention was conducted over approximately eight weeks, spanning from 25/03/2023 to 30/05/2023 during the second semester of the 2023 Ethiopian academic year. In the syllabus it was shown that the unit needed 24 periods to be accomplished. The EG was taught using GIBLEI approach, whereas the TLEI approach was employed for the CG. With the support of the researchers and laboratory technicians, the selected biology teachers delivered the following sub-topics from Unit 4 of the Grade 10 Biology textbook to both the EG and the CG. These were the organs of a flowering plant and the leaf, photosynthesis, transport in plants, and response in plants.

#### 2.4.2. Treatment procedure.

The intervention began after all preparatory tasks were finished. The procedures listed below were applied to every GIBLEI. The procedure was modified based on Blanchard et al [24]. With the lab technician’s assistance, the chosen teacher guides every step of the process. The following procedures were followed for all the 19 practical activities.

1) A week prior to each class, students were given semi-structured problems from unit 4 of the biology curriculum for grade 10.
2) The students used to look for an experimental procedure and plan their experiments for every problem until the following lab practice.
3) They used the results of their search to determine the layout of their experiment.
4) The students used to share their ideas and discuss their experimental design with other groups. At this point, they would go over every step of the experiment, the materials, and the rationale behind the materials and procedure they had selected.
5) The lab technician and the teacher have supplied the materials needed for the experiments. The students would then carry out the experiments in accordance with their design, taking notes on their observations in order to draw conclusions.
6) In relation to the theoretical sections, the students were expected to present to the class the conclusions they drew from their observations and experimental data.
7) After conducting the experiment, the groups attempted to respond to the questions relating to the experiment and the conclusions have been discussed in the classroom.
8) Assessments were carried out following each experiment to determine the extent to which students understood theoretical and scientific information relating to the lessons or experiments carried out.

Furthermore, the selected teacher used to prepare daily lesson plans by using the phases of the 5E lesson plan format for the EG. Engage, explore, explain, elaborate, and evaluate are the phases. For each lesson, all the above steps were structured in to this format. The majority of the guided inquiry based laboratory activities were incorporated in to the exploration phase for the EG. The Control Group (CG) was taught the same topics using the Traditional Laboratory Experiments Enriched Instruction (TLEI) approach, which primarily involved lecture-based teaching and teacher demonstrations. In this method, students acted as passive observers and relied on their textbooks. This approach differed from that used for the Experimental Group (EG) (see Table 1).During the intervention, the researchers used to carry out meetings with the selected teachers at each week ends to discuss on how to prepare daily lesson plan and how to implement the activities of the intervention in the treatment. At the end of the intervention, a post-test was administered to all students in the two groups**.** Moreover, classroom observation was carried out by the researchers using checklist during the intervention to evaluate whether the instruction was delivered according to the plan or not. The researchers also used to take notes and spent some time to interact with the students, as a participant observer.

**Table 1:** Comparison of GIBLEI and TLEI approaches across the 8-step intervention framework.

Step	GIBLEI (Guided Inquiry-Based Labs)	TLEI (Traditional Labs)	Similarity or Difference
1. Pre-Class Problem/ Task	Students were given semi-structured problems a week before class to plan inquiry activities.	No pre-class tasks or student planning; the teacher prepared demonstrations.	❌ Different
2. Planning Experiments	Students researched and planned their own experiments based on the given problems.	Not applicable; students were not involved in planning experiments.	❌ Different
3. Designing Experimental Layout	Students determined the materials and procedure for their designed experiments.	Not applicable; design was predetermined by the teacher.	❌ Different
4. Peer Discussion on Design	Students discussed experimental designs, materials, and rationale with peers before implementation.	Not applicable; no design discussion took place among students.	❌ Different
5. Performing the Experiment	Students performed the experiments they designed using supplied materials and made observations.	Teacher demonstrated the experiments; students observed and took notes.	❌ Different
6. Presentation of Findings	Students presented and discussed their findings and conclusions in class.	Teacher explained findings and conclusions; students completed worksheets.	❌ Different
7. Reflection and Discussion	Students answered follow-up questions and engaged in class discussions about outcomes.	Limited student discussion; primarily teacher-led explanation.	❌ Different
8. Assessment and Feedback	Formative assessments followed each experiment to assess conceptual and scientific understanding.	Assessment primarily through worksheets or oral questioning; no direct engagement with experimental understanding.	✔ Similar in intent, ❌ different in execution
9. Number of Practical Activities	19 experiments	19 experiments	✔ Same

### 2.5. Data analysis

Before conducting statistical analyses, the data were assessed to determine whether it satisfied the assumptions for parametric or non-parametric tests. Normality was evaluated using skewness and kurtosis values, where acceptable ranges were set between −2 and +2 [25]. Homogeneity of variance was checked using Levene’s test. The results indicated that all variables followed a normal distribution. Tests for homogeneity showed equal variances (p > 0.05) for pretest scores, posttest scores by gender, and the attitude dimension “Biology as a career” (Dimension-4) between the EG and the CG. However, significant differences in variances (p < 0.05) were found in the posttest results for overall attitude and other dimensions, including enthusiasm (Dimension-1), biology learning as a subject (Dimension-2), and biology as a process (Dimension-3).

Based on these findings, appropriate parametric tests were employed. For comparisons involving unequal variances, Welch’s t-test was used, while independent samples t-tests and paired samples t-tests were applied where variances were equal. Moreover, effect sizes were calculated using Cohen’s d, which quantifies the magnitude of group differences and provides practical significance beyond p-values [26]. Among various measures, Cohen’s d, eta squared (η²), and partial eta squared (ηₚ²), the choice depends on the analysis type. Cohen’s d is most suitable for t-tests, as in this study, where two group means and few dependent variables are compared. Interpreted at 0.2, 0.5, and 0.8 for small, medium, and large effects, it offered a simple and appropriate measure for this design [27].

Pearson correlation analyses were also conducted to examine the relationships among students’ posttest scores for overall biology attitudes and four subscales (enthusiasm toward biology, Biology learning as a subject, biology as a process, and biology as a future vocation) separately for the EG and the CG. All statistical analyses were performed using SPSS version 22, a widely recognized software for social science and educational research. SPSS was selected because it allows for easy implementation of parametric tests, Welch’s t-test, paired and independent samples t-tests, effect size calculations, and correlation analyses, ensuring robust and reliable results.

### 2.6. Ethics statement

This study was approved by the Research and Review Ethics Committee of Hawassa University (Reference No. CNCS-REC029/22). Following approval, the Department of Biology issued an official letter (Reference No. Bio/781/16), which we submitted to the North Shoa Zone Education Office together with an explanation of the study’s academic purpose. After receiving authorization from the office, the study was carried out with written informed consent obtained from school directors, teachers, and participating students. All signed consent forms were securely archived along with the collected data. Importantly, the study did not involve minors, as all student participants were from the same age group considered adults within the community.

## 3. Results of the study

### 3.1. Pretest scores

The pretest results show that there were no statistically significant differences between the EG and CG in their overall attitude toward biology or in any of the four attitude dimensions (enthusiasm, biology learning, biology as a process, and biology as a career), as indicated by the independent samples t-tests (see Table 2). This suggests that both groups had comparable baseline attitudes toward biology prior to the intervention, ensuring the initial equivalence of the groups. Table 2 further explores gender differences within each instructional group. In the EG, no significant gender differences were found across the overall attitude and the four dimensions. However, in the CG, significant gender differences were observed in overall attitude (p = .027), biology learning (p = .021), and biology as a career (p = .005), with female students scoring higher than male students in these areas. These findings indicate that, although the groups were generally equivalent at baseline, some gender-based differences existed within the CG before the intervention.

**Table 2:** Comparison of pretest scores of students’ overall attitudes and dimensions b/n groups and by gender.

Dependent variable	Group	N	Mean	SD	t	df	p-value
Overall attitude	EG	46	3.96	.49	−1.18	73	.241
	CG	29	4.09	.37			
	EG (Male)	22	3.88	.51	−1.14	44	.261
	EG (Female)	24	4.04	.48			
	CG (Male)	13	3.92	.42	−2.34	27	.027*
	CG (Female)	16	4.23	.26			
Enthusiasm	EG	46	4.07	.72	−1.62	73	.108
	CG	29	4.32	.49			
	EG (Male)	22	4.05	.70	−.14	44	.880
	EG (Female)	24	4.08	.76			
	CG (Male)	13	4.27	.47	−.48	27	.633
	CG (Female)	16	4.36	.53			
Biology learning	EG	46	3.99	.67	−.80	73	.426
	CG	29	4.11	.53			
	EG (Male)	22	3.85	.65	−1.43	44	.158
	EG (Female)	24	4.13	.67			
	CG (Male)	13	3.86	.55	−2.45	27	.021*
	CG (Female)	16	4.31	.43			
Biology as a process	EG	46	3.82	.61	−.77	73	.441
	CG	29	3.93	.61			
	EG (Male)	22	2.70	1.92	−2.36	44	.022*
	EG (Female)	24	3.75	.97			
	CG (Male)	13	3.98	.78	.35	27	.732
	CG (Female)	16	3.90	.45			
Biology as a career	EG	46	3.99	.63	−.37	73	.709
	CG	29	4.04	.65			
	EG (Male)	22	3.90	.57	−.91	44	.366
	EG (Female)	24	4.07	.68			
	CG (Male)	13	3.68	.56	−3.00	27	.005*
	CG (Female)	16	4.34	.58			

### 3.2. Overall attitude and its dimensions scores

The EG, taught using the Guided Inquiry-Based Laboratory Experiments Enriched Instructional (GIBLEI) approach, achieved a higher overall post-test attitude mean score (M = 4.60, SD = 0.19) than the Control Group (CG) taught with Traditional Laboratory Experiments Enriched Instructional (TLEI) (M = 4.06, SD = 0.37) (see Table 3). The EG’s smaller standard deviation indicates consistent positive attitudes, while the CG’s larger variation suggests less uniform engagement. A Welch’s t-test confirmed the difference was statistically significant (t = 7.324, p = 0.000014) with an extremely large effect size (Cohen’s d = 1.99). Consequently, the first null hypothesis (H₀₁) was rejected for overall attitude toward biology.

**Table 3:** Comparison of posttest mean scores of overall attitude and its dimensions between EG and CG.

Variable	Group	N	Mean	SD	T	p-value	d
Overall attitude	EG	46	4.60	.18	7.32	0.000014	1.99
	CG	29	4.06	.36			
Enthusiasm	EG	46	4.88	.14	5.84	0.000002	1.69
	CG	29	4.30	.52			
Biology learning as a subject	EG	46	4.37	.34	3.77	0.002	0.89
	CG	29	3.94	.65			
Biology as a process	EG	46	4.90	.09	12.58	1.7154E-13	3.68
	CG	29	3.97	.39			
Biology as a career	EG	46	4.32	.41	1.81	.074	0.43
	CG	29	4.12	.52			

The posttest attitude scores of the EG and CG were examined across four affective dimensions: Enthusiasm toward Biology, Biology Learning, Biology as a Process, and Biology as a Career. A Welch’s t-test (unequal variance t-test) was conducted to assess differences in post-test mean scores of the first three attitude dimensions (Enthusiasm, Biology learning as a subject, and Biology as a process) between the EG and CG, given that the assumption of homogeneity of variances was violated. Meanwhile, the differences in the posttest mean scores for attitude dimension 4 (Biology as a career) between EG and CG was also examined by using the independent samples t-test (Table 3). The results revealed that the EG consistently outperformed the CG in the first three dimensions, demonstrating the multifaceted effectiveness of the GIBLEI approach. In the Enthusiasm dimension, the EG reported a significantly higher mean score (M = 4.88, SD = 0.14) compared to the CG (M = 4.30, SD = 0.52). The difference was statistically significant (t = 5.842, p = 0.000002), with a very large effect size (d = 1.69), suggesting that the GIBLEI approach fostered both strong and consistent enthusiasm among students in the EG. For Biology Learning, the EG also achieved a higher mean score (M = 4.37, SD = 0.34) than the CG (M = 3.94, SD = 0.65). The difference was statistically significant (t = 3.77, p = 0.002), with a large effect size (d = 0.89), indicating that the approach helped improve students’ attitudes toward biology as a subject.

The most significant outcome was observed in the Biology as a Process dimension. The EG scored markedly higher (M = 4.90, SD = 0.09) than the CG (M = 3.97, SD = 0.39). This difference was highly significant (t = 12.58, p = 1.7154E-13) and was associated with an exceptionally large effect size (d = 3.68). These results suggest a deep epistemological shift in how EG students view biology, as an evolving, inquiry-driven discipline.

In contrast, for Biology as a Career, although the EG mean score (M = 4.32, SD = 0.41) was higher than that of the CG (M = 4.12, SD = 0.52), the difference was not statistically significant (t = 1.81, p = 0.074), and the effect size was small (d = 0.43), indicating that career aspirations may be shaped by broader external influences beyond classroom instruction. Accordingly, the second null hypothesis (H₀₂) was rejected for the first three dimensions, but retained for the fourth.

Moreover, Table 4 presents the Pearson correlations among posttest scores for overall biology attitudes and attitude dimensions in the GIBLEI and TLEI groups, respectively. In both groups, Overall Biology Attitude showed strong positive correlations with Enthusiasm toward biology, Biology learning as a subject, and Biology as a future vocation. The correlations were slightly stronger in the EG, particularly between Overall biology attitude and biology learning as a subject(r = .806) and Biology as a future vocation (r = .825). Enthusiasm toward biology was positively associated with Biology learning and future vocation in both groups, but not significantly related to Biology as a process. Across both groups, Biology as a process showed weak and mostly non-significant correlations with other variables. These findings suggest that GIBLEI instruction fostered stronger and more interconnected positive attitudes toward biology compared to the traditional TLEI approach.

**Table 4:** Pearson correlations among posttest scores for overall attitudes and attitude dimensions in the EG and CG.

Experimental group (N = 46)						Control group (N = 29)				
1	2	3	4	5	Variables	1	2	3	4	5
	.509**	.806**	.333*	.825**	1. Overall attitude		.793**	.812**	.273	.740**
		.333*	.216	.315*	2. Enthusiasm			.582**	.093	.483**
			.160	.398**	3. Biology Learning				−.124	.385*
				.163	4. Biology as a Process					.140
					5. Biology as a career					

**Note:** *p < .05, **p < .01 (2-tailed).

### 3.3. Pretest vs. Post-test analysis

The analysis demonstrated a significant and meaningful improvement in students’ attitudes toward biology after participating in the GIBLEI approach. The EG showed a notable increase in mean attitude scores from 3.96 (SD = 0.49) on the pretest to 4.60 (SD = 0.18) on the posttest (see Table 5). This improvement was statistically significant, as indicated by a paired-samples t-test (t = –8.74, p = 2.9157E-11), and was accompanied by a very large effect size (d = 1.29), reflecting a strong practical impact. Additionally, the reduced posttest standard deviation suggests that the positive shift was experienced consistently across most students, highlighting the widespread effectiveness of the GIBLEI model.

**Table 5:** Comparison of BAQ pre and posttests mean scores in both the EG and CG.

Group	Test	N	Df	T	p-value	d
EG	Pretest	46	45	−8.74	2.9157E-11	1.29
	Post-test	46				
CG	Pretest	29	28	.495	.624	0.09
	Post-test	29				

In contrast, the Control Group (CG), which followed the TLEI approach, showed no significant change. Their mean scores remained almost unchanged from pretest (M = 4.09, SD = 0.37) to posttest (M = 4.06, SD = 0.36), with a non-significant t-test result (t = 0.495, p = .624) and a negligible effect size (d = 0.09). This led to the rejection of the third null hypothesis (H₀₃) for the EG.

### 3.4. Gender analysis

The gender analysis revealed significant differences in student attitudes between male and female students within the CG. At the pretest stage, females outperformed males in overall attitude (M = 4.23 vs. M = 3.92, p = .027), biology learning (M = 4.31 vs. M = 3.86, p = .021), and biology as a career (M = 4.34 vs. M = 3.68, p = .005). These differences persisted into the posttest, where females continued to score higher than males in overall attitude (M = 4.19 vs. M = 3.89, p = .025). In contrast, the EG showed no significant gender differences in overall attitude at pretest (p = .261), indicating a relatively balanced starting point between male and female students (see Table 6).

**Table 6:** Comparison of male and female students’ post-test attitudes toward biology within the EG and CG across the overall and the four attitude dimensions (independent samples t-tests).

Test	Dependent variables	Group	Gender	N	Mean	SD	t	Df	p-value
Post-test	Overall attitude	EG	M	22	4.58	.18	−.78	44	.439
			F	24	4.62	.18			
		CG	M	13	3.89	.36	−2.36	27	.025
			F	16	4.19	.32			
	Enthusiasm	EG	M	22	4.83	.16	−2.24	44	.030
			F	24	4.92	.11			
		CG	M	13	4.12	.56	−1.71	27	.099
			F	16	4.44	.46			
	Biology learning	EG	M	22	4.33	.29	−.80	44	.423
			F	24	4.41	.38			
		CG	M	13	3.67	.77	−2.04	27	.052
			F	16	4.15	.46			
	Biology as a process	EG	M	22	4.90	.07	.21	44	.830
			F	24	4.89	.10			
		CG	M	13	3.95	.38	−.19	27	.847
			F	16	3.98	.40			
	Biology as a career	EG	M	22	4.26	.42	−.836	44	.407
			F	24	4.37	.40			
		CG	M	13	3.93	.40	−1.821	27	.080
			F	16	4.27	.57			

Posttest results for the EG showed that male and female students maintained statistically similar attitudes toward biology (p = .439), with females (M = 4.62) slightly ahead of males (M = 4.58). A small but significant difference was noted in enthusiasm, favoring females (M = 4.92 vs. M = 4.83, p = .030). However, no statistically significant gender differences were observed in biology learning (p = .423), biology as a process (p = .830), or biology as a career (p = .407), suggesting balanced posttest outcomes across these dimensions. In the CG, gender differences persisted or approached significance, with females continuing to score higher than males in enthusiasm (M = 4.44 vs. M = 4.12, p = .099), biology learning (M = 4.15 vs. M = 3.67, p = .052), and biology as a career (M = 4.27 vs. M = 3.93, p = .080). This resulted in the rejection of hypothesis four (H_04_) for the EG.

## 4. Discussion of the findings

The present study examined the effects of the Guided Inquiry-Based Laboratory Experiments Enriched Instructional (GIBLEI) approach on secondary school students’ attitudes toward biology in Ethiopia, comparing it with the Traditional Laboratory Experiments Enriched Instructional (TLEI) approach. One of the most striking outcomes was the substantial improvement in overall post-test attitudes among students taught with the GIBLEI approach compared to those in the TLEI group. Students in the EG demonstrated a mean post-test attitude of 4.60 (SD = 0.19), significantly higher than the CG’s mean of 4.06 (SD = 0.37), with a very large effect size (Cohen’s d = 1.99). According to Lakens [28], an effect size greater than 0.80 is considered large, and values above 1.00 reflect a highly meaningful and practically significant difference in real world educational settings. The relatively small standard deviation in the EG also suggests that these benefits were consistent across students, whereas the greater variation in the CG indicates that TLEI failed to promote uniformly positive attitudes.

The findings align with constructivist learning theory, which emphasizes that meaningful learning occurs when learners actively construct knowledge through interactive, context-rich, and socially mediated experiences [29]. GIBLEI’s emphasis on student inquiry, collaboration, and reflection appears to have fostered a stimulating classroom environment, supporting broad-based attitudinal gains. This result also resonates with Self-Determination Theory [30], which posits that positive attitudes and motivation emerge when learners’ needs for autonomy, competence, and relatedness are satisfied. GIBLEI’s structured flexibility likely fulfilled these needs by providing students with a sense of control, confidence in their inquiry skills, and collaborative interactions. In contrast, the TLEI approach, being largely teacher-centered and procedural, offered minimal opportunities for these motivational experiences, explaining the stagnation of attitudes in the control group. Beyond theoretical support, these results have practical significance. In contexts like Ethiopia, where didactic teaching predominates, adopting inquiry-based strategies has the potential to enhance not only students’ understanding of biology but also their emotional and motivational connection to science. Positive attitudes are essential for sustaining engagement and for nurturing future scientists, educators, and informed citizens.

A deeper examination of specific affective dimensions revealed that GIBLEI’s impact was multidimensional, extending across enthusiasm toward biology, perceptions of biology learning, and understanding biology as a scientific process. In terms of enthusiasm, students in the EG reported very high engagement (M = 4.88, SD = 0.14), significantly exceeding the control group (M = 4.30, SD = 0.52) with a large effect size (d = 1.69). The consistency of high enthusiasm scores suggests that the inquiry-based approach cultivated a uniformly engaging classroom culture. This finding aligns with research showing that inquiry-based instruction enhances students’ excitement, curiosity, and enjoyment in learning science [31]. By actively involving students in hands-on exploration, questioning, and collaborative problem-solving, GIBLEI transformed the biology classroom into a space where students felt personally invested in learning. Conversely, the traditional laboratory approach produced lower and more variable enthusiasm, reflecting its procedural rigidity and passive learning structure, which tend to diminish opportunities for curiosity and discovery.

Similarly, attitudes toward biology as a subject were significantly more positive among GIBLEI students (M = 4.37, SD = 0.34) than in the control group (M = 3.94, SD = 0.65), with a large effect size (d = 0.89). This suggests that inquiry-based learning enhanced students’ perceptions of biology, making it more valuable, relevant, and appealing. The contextualization of biology content through active investigation allowed students to develop a sense of ownership over their knowledge and recognize the subject’s relevance to real-life contexts. This outcome supports constructivist principles and corroborates findings from other studies reporting that inquiry-based methods improve students’ engagement with science subjects [32].

The most pronounced effect, however, was observed in attitudes toward biology as a process of scientific inquiry. Students in the experimental group achieved near-perfect scores (M = 4.90, SD = 0.09), compared with the control group (M = 3.97, SD = 0.39), producing an extraordinarily large effect size (d = 3.68). This demonstrates that GIBLEI powerfully influenced students’ epistemological understanding, helping them appreciate biology as a dynamic and investigative discipline. These findings align with international reform initiatives emphasizing the importance of scientific practices and epistemic understanding in science education [33]. Understanding science as a process rather than as a static body of facts is central to scientific literacy, which equips learners to engage critically with real-world issues. GIBLEI’s structured inquiry activities, including hypothesis generation, experimentation, and reflection, effectively nurtured this process-oriented perspective.

In contrast to these strong effects on engagement and epistemological understanding, attitudes toward biology as a career showed no statistically significant difference between groups. Although experimental group students scored slightly higher (M = 4.32, SD = 0.41) than control group students (M = 4.12, SD = 0.52), the effect size was small (d = 0.43) and the difference non-significant (t = 1.81, p = .074). This outcome suggests that while GIBLEI effectively enhanced classroom engagement and subject appreciation, it did not substantially influence career aspirations. Career choices are shaped by a complex interplay of socio-cultural, economic, and familial factors [34]. In resource-limited settings such as Ethiopia, structural barriers, including limited job opportunities, family expectations, and societal perceptions, can overshadow the influence of classroom-based interventions. This finding highlights the importance of complementing pedagogical reforms with career guidance programs, exposure to role models, and systemic support to meaningfully impact students’ professional aspirations in science.

Pretest-posttest comparisons further illustrate GIBLEI’s effectiveness. Students in the experimental group improved from a pretest mean of 3.96 to a posttest mean of 4.60, with a large effect size (d = 1.29). Notably, the reduced variability at posttest suggests that GIBLEI produced equitable gains, benefiting learners across the spectrum of initial attitudes. By contrast, the control group exhibited stagnant scores (M = 4.09 pretest to M = 4.06 posttest), highlighting the limitations of TLEI in shifting affective orientations. Traditional, teacher-centered methods are less capable of inspiring curiosity or motivation, consistent with prior research [1]. These results underscore the transformative potential of inquiry-based pedagogy in achieving meaningful attitudinal growth.

A significant contribution of this study lies in the analysis of gender-based outcomes. At baseline, gender disparities were evident in the control group, with female students outperforming males in overall attitude, biology learning, and career aspirations. These gaps persisted at posttest, suggesting that TLEI not only failed to address gender inequalities but may have reinforced them. Male students in the control group even showed declines in enthusiasm and learning attitudes, highlighting the risk of disengagement under passive instructional methods. In contrast, the experimental group exhibited far more equitable outcomes. No significant gender differences were observed at pretest, and at posttest, males and females scored similarly across overall attitude, biology learning, process understanding, and career aspirations. The only exception was in enthusiasm, where females scored slightly higher (M = 4.92 vs. 4.83). Both genders, however, achieved substantial pre-to-post gains, with male students showing particularly large improvements in appreciation of biology as a process (+2.20). These findings indicate that GIBLEI not only enhances overall attitudes but also re-engages male students who might otherwise be alienated by traditional methods. This aligns with research suggesting that inquiry-based instruction promotes gender equity by fostering inclusive, collaborative, and dialogic classroom dynamics [35]. Slightly higher female enthusiasm is consistent with studies indicating that female students may be more responsive to relational and interactive pedagogies [34]. Overall, GIBLEI appears to support equity and inclusion while leveraging natural differences in affective responsiveness.

Correlation analyses provided deeper insights into students’ attitudes. In both the experimental and control groups, overall attitudes were strongly associated with enthusiasm, biology learning, and career aspirations, but only weakly related to understanding biology as a process. This suggests that students generally find biology engaging, relevant, and professionally meaningful, yet their grasp of biology as a scientific process is somewhat disconnected from their emotional and motivational orientations. This indicates a conceptual-emotional gap, whereby learners may intellectually recognize scientific inquiry without integrating this understanding into personal motivation or meaning, a phenomenon documented in previous research [36]. These findings suggest a need for instructional strategies that explicitly link epistemological comprehension with personal relevance to enhance engagement and long-term attitudinal impact. The experimental group, however, exhibited stronger positive interconnections among sub-dimensions, reflecting a holistic and integrated attitudinal profile. In contrast, the control group showed weak or even negative correlations involving biology as a process, indicating fragmented attitudes. These results suggest that GIBLEI not only enhances individual dimensions of attitude but also helps students integrate them into a cohesive affective framework, a characteristic of expert-like science attitudes [37]. Traditional methods, by contrast, appear to foster compartmentalized attitudes that may undermine long-term engagement and epistemological appreciation.

This study contributes to the literature by providing evidence from Ethiopian secondary schools, a context often underrepresented in research on inquiry-based learning. While prior studies have documented the cognitive and affective benefits of inquiry-based approaches in higher education and developed contexts [38], this research demonstrates that such approaches can produce transformative outcomes even in resource-limited settings. The findings extend international knowledge on inquiry-based pedagogy and offer a model for developing countries seeking to reform science education, showing that with appropriate instructional design, students can achieve substantial gains in engagement, learning, and appreciation of science as a process. Overall, the study underscores the potential of GIBLEI to foster meaningful, equitable, and integrated attitudinal growth, suggesting that inquiry-based strategies should be prioritized in both policy and practice to support the development of scientifically literate, motivated, and inclusive learner populations.

## 5. Conclusions and implications

### 5.1. Conclusions

This study set out to examine the effects of the GIBLEI approach on secondary school students’ attitudes toward biology in comparison with the TLEI method. The findings consistently demonstrated that GIBLEI produced significantly more positive attitudes, particularly in enthusiasm, perceptions of biology learning, and understanding biology as a process, while TLEI showed little or no impact. Importantly, GIBLEI fostered equitable attitudinal gains across genders and re-engaged male students who were at risk of disengagement under traditional methods. However, neither approach produced a substantial change in students’ career aspirations, reflecting the influence of broader socio-cultural and economic factors.

Collectively, these results suggest that inquiry-based pedagogy is more effective than traditional teacher-centered instruction in cultivating positive, integrated, and inclusive attitudes toward biology. By creating opportunities for autonomy, collaboration, and reflection, GIBLEI not only enhanced students’ immediate engagement but also strengthened their epistemological appreciation of biology as an investigative science. The limited effect on career attitudes highlights the need for complementary initiatives such as career guidance and systemic support.

Theoretically, the study contributes to science education research by documenting the “conceptual–emotional gap,” wherein students’ understanding of biology as a process does not always translate into motivation or personal meaning. Practically, the findings highlight the potential of inquiry-based instruction to transform classrooms in resource-limited settings like Ethiopia, advancing both equity and scientific literacy. These insights provide evidence to support educational policy reforms that prioritize inquiry-based approaches and point to future research exploring strategies to better connect epistemological understanding with personal motivation and career pathways.

### 5.2. Implications

The findings of this study provide strong evidence that the GIBLEI approach is effective in enhancing secondary school students’ attitudes toward biology. Significant improvements in enthusiasm, perception of biology as a course, and understanding of biology as a process highlight its potential to foster active engagement, curiosity, and deeper learning. These outcomes suggest that integrating GIBLEI activities into secondary school biology curricula at both national and institutional levels could create a more interactive and student-centered learning environment. To ensure successful implementation, teacher professional development is essential, with emphasis on collaborative inquiry, experimental design, and facilitation of reflective discussions.

Nevertheless, the study revealed that GIBLEI had limited influence on students’ perceptions of biology as a career. This limitation underscores the need for complementary interventions, such as career talks by professionals, field visits, mentorship, and structured career guidance, to expand students’ awareness of biology-related career pathways. When integrated with such strategies, GIBLEI could not only improve classroom engagement but also positively influence long-term career aspirations.

The gender-neutral outcomes observed in this study suggest that GIBLEI can play an important role in promoting equity in science education. By reducing gender disparities in participation and achievement, it ensures that both male and female students benefit equally from inquiry-based learning. This makes GIBLEI a valuable pedagogical approach for fostering inclusion and broadening participation in STEM fields.

For policymakers and stakeholders, prioritizing GIBLEI means allocating resources to improve laboratory facilities, developing teacher training programs, and ensuring equitable access to inquiry-based methods in resource-limited contexts. Importantly, this proposal should also be considered for application in other areas of the natural sciences, such as chemistry, physics, and earth science, where guided inquiry-based laboratory experiences can similarly enhance students’ engagement, conceptual understanding, and process skills.

Future research should explore the long-term impact of GIBLEI on students’ learning outcomes, academic progress, and career choices across various science disciplines. By addressing these dimensions, GIBLEI has the potential to advance science education more broadly and inspire the next generation of scientists, innovators, and problem-solvers.

### Limitations

Variations in resource availability and teacher-related variables could have influenced the research outcomes. Conducting the study in natural school environments, where classes were organized by administrators, may also limit the generalizability of the results to other settings. In addition, the relatively small sample size of 75 students restricts the extent to which the findings can be generalized. The study was also limited to only two schools, both located in urban areas. Including a more diverse sample, particularly schools from both rural and urban contexts, would strengthen the robustness and applicability of the results.

To address these limitations and enhance the validity of future research, several strategies can be considered. These include standardization of resources, enhanced teacher training and monitoring, implementation of randomized controlled trials, longitudinal studies to track long-term effects, expansion of the sample to include more schools across varied settings, and collaborative research partnerships with stakeholders to ensure alignment with educational priorities. By implementing these strategies, researchers can overcome the identified limitations and contribute to advancing knowledge on effective instructional approaches in biology education.
